# Haplotype Explorer: an infection cluster visualization tool for spatiotemporal dissection of the COVID-19 pandemic

**DOI:** 10.1093/g3journal/jkab126

**Published:** 2021-04-23

**Authors:** Tetsuro Kawano-Sugaya, Koji Yatsu, Tsuyoshi Sekizuka, Kentaro Itokawa, Masanori Hashino, Rina Tanaka, Makoto Kuroda

**Affiliations:** Pathogen Genomics Center, National Institute of Infectious Diseases, Tokyo, Japan

**Keywords:** SARS-CoV-2, COVID-19, Haplotype network, Infection clusters, Infectious diseases, epidemiology

## Abstract

**Summary:**

Many of software for network visualization are available, but existing software have not been optimized to infection cluster visualization, especially the current worldwide invasion of COVID-19 since 2019. To reach the spatiotemporal understanding of epidemics, we have developed Haplotype Explorer. In Haplotype Explorer, users can explore the network interactively with metadata like accession number, locations, and collection dates. Time dependent transition of the network can be exported as continuous sections for making a movie. Here, we introduce features and products of Haplotype Explorer, demonstrating time-dependent snapshots and a movie of haplotype networks inferred from total of 4,282 SARS-CoV-2 genomes.

**Abstract:**

The worldwide eruption of COVID-19 that began in Wuhan, China in late 2019 reached 10 million cases by late June 2020. In order to understand the epidemiological landscape of the COVID-19 pandemic, many studies have attempted to elucidate phylogenetic relationships between collected viral genome sequences using haplotype networks. However, currently available applications for network visualization are not suited to understand the COVID-19 epidemic spatiotemporally due to functional limitations, that motivated us to develop Haplotype Explorer, an intuitive tool for visualizing and exploring haplotype networks. Haplotype Explorer enables to dissect epidemiological consequences via interactive node filters and provides the perspective on infectious disease dynamics depend on regions and time, such as introduction, outbreak, expansion, and containment. Here, we demonstrate the effectiveness of Haplotype Explorer by showing features and an example of visualization. The demo using SARS-CoV-2 genomes are available at https://github.com/TKSjp/HaplotypeExplorer/blob/master/Example/. There are several examples using SARS-CoV-2 genomes and Dengue virus serotype 1 E-genes sequence.

### Introduction

To control infectious diseases, it is important to quickly identify emerging infection clusters before they become critical issues. Many applications have been developed to assist researchers understanding the latest epidemiology. Indeed, the recent intensification of the COVID-19 pandemic, which began in late 2019 in Wuhan, China, has prompted development of new software to support investigations of this virus. For example, Nextstrain (Hadfield *et al*. 2018) is one of the most popular web services related to the COVID-19 pandemic which provides interactive molecular phylogenetic trees and geographic maps representing possible virus transmission routes. The COVID-19 Genome Tracker (Akther *et al*. 2020) is another unique application which shows the evolution of the SARS-CoV-2 using a haplotype network. This tool can dynamically display metadata, such as isolate conditions, locations, and mutations, compared to the reference genome. National Genomics Data Center in China also provides Viral Haplotype Network (https://bigd.big.ac.cn/ncov/haplotype/; Song *et al*. 2020). Although it is specialized for the COVID-19, the spatiotemporal eruption of that is visualized interactively.

So far, many phylogenetic trees and haplotype networks using the SARS-CoV-2 genome have been inferred because they are suited to interpret genetic and epidemiological relationships among sequences (Sekizuka *et al*. 2020a, b, and Giovanetti *et al*.). In this time, haplotype networks are especially useful due to their potential for displaying short-term diversification of closely related genomes. Many available software programs for network inferring, such as TCS (Clement *et al*. 2000), PopART (Leigh and Bryant 2015), and Network (Bandelt *et al*. 1999), have supported these studies using haplotype networks of the SARS-CoV-2. Although these applications also work as network viewers, several alternatives are also available for additional annotation and exploration, including Cytoscape (Su *et al*. 2014), Gephi (Bastian *et al*. 2009), and tcsBU (Múrias dos Santos *et al*. 2016).

However, currently available tools are sometimes not the best to visualize infection clusters because they usually do not simultaneously fulfill the requirements essential to dissect epidemic situations: 1) nodes that can be dynamically filtered with metadata by complex search queries, 2) nodes can be indicated by real-time pie charts, which reflect sample size and content proportions at a given time span, and 3) creating interactive distribution files which require no external software installation. Hence, we endeavored to develop Haplotype Explorer, a specialized network viewer which assists onsite actions against emerging pathogens. Haplotype Explorer is a novel platform for network analysis displaying the network data from small scale to large scale and helping users to dissect the network by complex metadata filters. It also can export not only figure at a certain timepoint but continuous sections for constructing a movie to help people understanding the expansion of the pathogen at a glance.

### Materials and Methods

#### Implementation and workflow

Haplotype Explorer is a JavaScript application executable in web browser, so it does not require uploading data to an external web server. This allows users to analyze confidential data securely. It can produce distributions in HTML, which enables users to share originated networks with others easily. The network structure is written in JavaScript Object Notation (JSON) format which can be generated automatically from a multi-FASTA file with the provided python programs (createHTML.py). The production of network (result.html) from the raw multi-FASTA file (input.fasta) is very straightforward like shown in Figure 2. In short, running "createHTML.py" will correct, curate, align sequences in input.fasta, execute TCS analysis, and convert data to an HTML file (result.html). We confirmed compatibilities of Haplotype Explorer and the bundled python scripts with the latest versions of Safari, Firefox, Edge, Chrome, and Python3 on macOS Catalina 10.15.3, respectively.

**Data analysis**

Whole genome sequences were retrieved from GISAID (Shu and McCauley 2017) on 9 June 2020 using the following options: 1) collection date was before 21 March 2020, 2) host was only human, 3) check was on for “complete,” “high coverage,” and “low coverage excl.” After retrieval of a total of 9,583 sequences, they were curated using several external software (*e.g*. removing low-quality sequences, such as those containing spaces, gaps, degenerated bases, and ambiguous collection dates (i.e., month or date are absent in collection date) using seqkit (Shen *et al*. 2016) and the Linux sed command) Passing sequences were aligned by MAFFT (Katoh *et al*. 2002), clustered by CD-HIT (Fu *et al*. 2012; threshold: 100% identical), and SNVs were extracted by snp-sites; Page *et al*. 2016). The TCS analysis was run using extracted SNVs, and the resultant GraphML (.gml) file was converted into JSON format which is compatible to Haplotype Explorer. In following analyses, we collected figures by applying the filters “~YYYYMMDD” from the initial day (31 December 2019; Wuhan-Hu-1) to 21 March 2020.

### Results and Discussion

#### Principal features of Haplotype Explorer toward epidemic dissection

The primary feature of Haplotype Explorer is a vibrant and interactive visualization function utilizing D3.js (Bostock *et al*. 2011) and metadata, including sample size, accession number, collected location, and collection date, which are important clues for understanding the epidemic. Each node is represented by differently sized pie charts calculated from sample number and location proportion described in the metadata. Nodes and related edges can be interactively highlighted when a specific node is left-clicked, making it easy to dissect a crowded network with large samples (Figure 1). Users can quickly look into the node of interest by zooming with the scroll-wheel, and show metadata by mousing-over the tool-tip window. The application has four text boxes for filtering nodes: Sequence ID, location, YYYYMMDD~, and ~YYYYMMDD. Filters can be combined, and the Sequence ID and location can be specified by regular expressions. The current view of the network can be exported in a JSON format file, and users can resume it by importing the JSON. Finally, the current SVG view can be converted into a high-resolution PNG image using the export button. We also provide python scripts for assisting haplotype network construction with in-house data. Details are shown in the flow-diagram (Figure 2).

**Demonstration of Haplotype Explorer: spatiotemporal dissection of multimodal epidemics**

The epidemic context of the SARS-CoV-2 from January 1 to March 21 was visualized by Haplotype Explorer (Figure 3). We began by capturing a snapshot for 21 March 2020 as an overall view. Haplotype Explorer effectively discerned significantly large, but distinctly invaded clusters consisting of a dozen to over one hundred genome collections formed by late March (Figure 3A; magenta arrowhead). In order to understand epidemics in a time-dependent manner, Haplotype Explorer can also generate snapshots for specified dates (Figure 3B).

**Data Availability**

All DNA sequences used in this study were downloaded from the GISIAD.

Source codes are available from the following URL: https://github.com/TKSjp/HaplotypeExplorer/. Supplemental Material available at figshare: https://doi.org/10.25387/g3.14349563.

### Author contributions

Manuscript preparation: TKS MK

Data analysis: TKS MK KY TS

Data collection: KI MH RT

### Acknowledgements

We would like to thank Editage (www.editage.com) for English language editing.

**Funding**: This study was supported by a Grant-in Aid from the Japan Agency for Medical Research and Development (AMED) under Grant number JP19fk0108103, JP19fk0108104, and JP20fk0108063. The funding agencies had no role in the study design, data collection or analysis, decision to publish, or manuscript preparation. We would like to thank all authors who have deposited genome sequences in the GISAID EpiCoV Database.

**Conflict of Interest**: none.

| | **Haplotype Explorer** | tcsBU | COVID-19 Genome Tracker | Cytoscape | Gephi | PopART | TCS |
|---|---|---|---|---|---|---|---|
| Generate explorable HTML distribution | ✓ | | | | | | |
| Time-dependent node pie charts | ✓ | | | | | | |

| | | | | | | | |
|---|---|---|---|---|---|---|---|
| Filter by metadata | ✓ | | ✓ | ✓ | ✓ | ✓ | |
| Resume operation | ✓ | | | ✓ | ✓ | ✓ | ✓ |
| Export image | ✓ | ✓ | | ✓ | ✓ | ✓ | ✓ |
| Use in-house data | ✓ | ✓ | | ✓ | ✓ | ✓ | ✓ |
| time orchestration of node positions | ✓ | ✓ | ✓ | | ✓ | | |
| Run on web browser | ✓ | ✓ | ✓ | | | | |

**Table 1 | Comparison of features with other applications**

The significance of Haplotype Explorer is that it enables users to generate an explorable HTML distribution, which includes several features all in one. It requires no external software other than a modern web browser to open the network, making it easy to share data. Furthermore, it can draw nodes as pie charts based on the specified span input into the search boxes, supporting spatiotemporal dissection of the network.

### Figure legends

**Figure 1 | Introduction of Haplotype Explorer.**

**(A)** The view of Haplotype Explorer **(B)** Introduction of features of Haplotype Explorer. It can modify distances and attractive forces among nodes and avoid overlapping automatically using the slide bar. Nodes can be moved by dragging. After manipulation of the appearance of the network, the view can be exported into PNG and JSON formats. Nodes are easily hidden or visible depending on keyword filters; accession ID, location, collection date from YYYYMMDD, and until YYYYMMDD. In cases where users specify dates, the pie chart is redrawn according to metadata so as to match to the queues. The metadata is displayed by mouse-hover, making it easy to inspect the node of interest.

**Figure 2 | The workflow and dependencies of Haplotype Explorer.**

Users can visualize own data in Haplotype Explorer by running bundled python program (createHTML.py). After preparation of "input.fasta", the python script automatically processes sequences, runs analysis, and produces the html file (result.html).

**Figure 3 | Demonstration of spatiotemporal analysis of the SARS-CoV-2 genomic network using Haplotype Explorer.**

(A) An example of the exported network generated by Haplotype Explorer using 1,729 of SNVs calculated from 4,282 of world-wide SARS-CoV-2 genomes until 21 March 2020 obtained from the GISAID database. Each node size depends on sample size, and node colors differ by locations. The black arrow indicates the Wuhan-Hu-1 reference sequence. Magenta arrows indicate that distinct erosion of COVID-19 cases has occurred mainly in Europe or America. (B) Three snapshots of the SARS-CoV-2 genomic network around Wuhan, China from January 1 to March 1. Haplotype Explorer enabled us to dissect a haplotype network depending on metadata, giving significant insights into the epidemic.

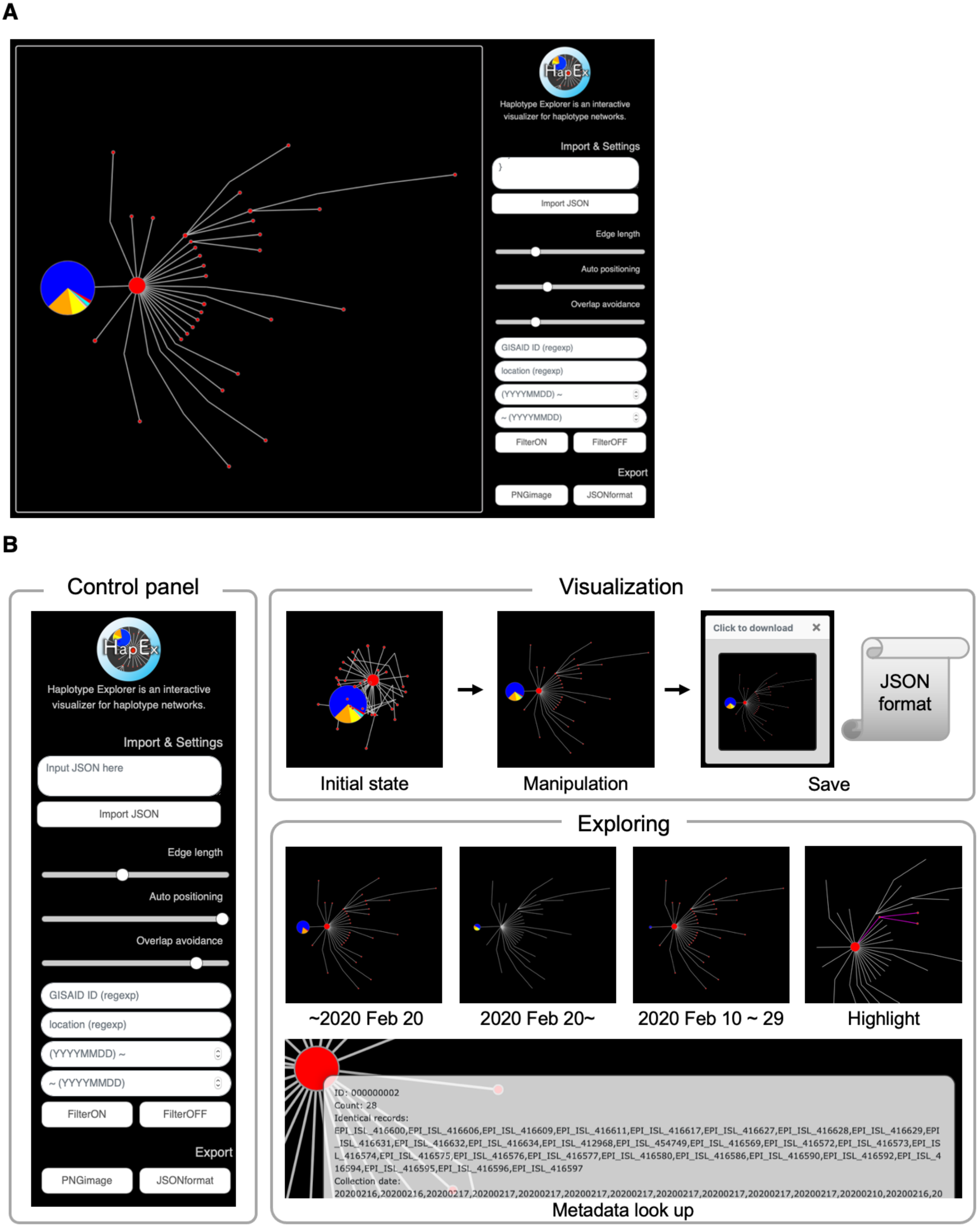

A
Haplotype Explorer is an interactive visualizer for haplotype networks.
Import & Settings
Import JSON
Edge length
Auto positioning
Overlap avoidance
GISAID ID (regexp)
location (regexp)
(YYYYMMDD) ~
~ (YYYYMMDD)
FilterON
FilterOFF
Export
PNGimage
JSONformat
B
Control panel
Haplotype Explorer is an interactive visualizer for haplotype networks.
Import & Settings
Input JSON here
Import JSON
Edge length
Auto positioning
Overlap avoidance
GISAID ID (regexp)
location (regexp)
(YYYYMMDD) ~
~ (YYYYMMDD)
FilterON
FilterOFF
Export
PNGimage
JSONformat
Visualization
Click to download
JSON format
Initial state
Manipulation
Save
Exploring
~2020 Feb 20
2020 Feb 20~
2020 Feb 10 ~ 29
Highlight
ID: 000000002
Count: 28
Identical records:
EPI_ISL_416600,EPI_ISL_416606,EPI_ISL_416609,EPI_ISL_416611,EPI_ISL_416617,EPI_ISL_416627,EPI_ISL_416628,EPI_ISL_416629,EPI_ISL_416631,EPI_ISL_416632,EPI_ISL_416634,EPI_ISL_412968,EPI_ISL_454749,EPI_ISL_416569,EPI_ISL_416572,EPI_ISL_416573,EPI_ISL_416574,EPI_ISL_416575,EPI_ISL_416576,EPI_ISL_416577,EPI_ISL_416580,EPI_ISL_416586,EPI_ISL_416590,EPI_ISL_416592,EPI_ISL_416594,EPI_ISL_416595,EPI_ISL_416596,EPI_ISL_416597
Collection date:
20200216,20200216,20200217,20200217,20200217,20200217,20200217,20200217,20200217,20200217,20200210,20200216,20
Metadata look up

## Haplotype Explorer

### View example data

Open Result_example.html

### View in-house data

**Install dependencies**

1. All depending programs other than network analyzing software (TCS) can be installed via Anaconda. If you do not have conda, download and install Anaconda (https://www.anaconda.com).
2. Open terminal and execute following command:
   `conda install -c bioconda seqkit mafft cd-hit snp-sites`
3. Download TCS from Softpedia (https://www.softpedia.com/get/Science-CAD/Posada-TCS.shtml)

**Retrieve sequences**

1. Create your account of GISAID (https://www.gisaid.org)
2. Log-in GISAID and open EpiCoV/Browse.
3. Specify “Host" as “Human” and check on for “complete”, “high coverage”, and “low coverage excl”.
4. Check on sequences of interest and download them as “input0.fasta”. (Note: currently GISAID restricts downloading over 10,000 records all at once)

***Step1.py**

- seqkit
- MAFFT
- snp-sites
- cd-hit-est
- *parseCdhit.py
- *makePhy.py

1. Open terminal and execute following command:
   `python3 Step1.py`

***Step2.py**

- TCS

1. Open terminal and execute following command: `python3 Step2.py`
2. Run TCS and export the result as “TCS.gml” in GraphML

***Step3.py**

1. Open terminal and execute following command:
   `python3 Step3.py`

Open Result_in-house.html

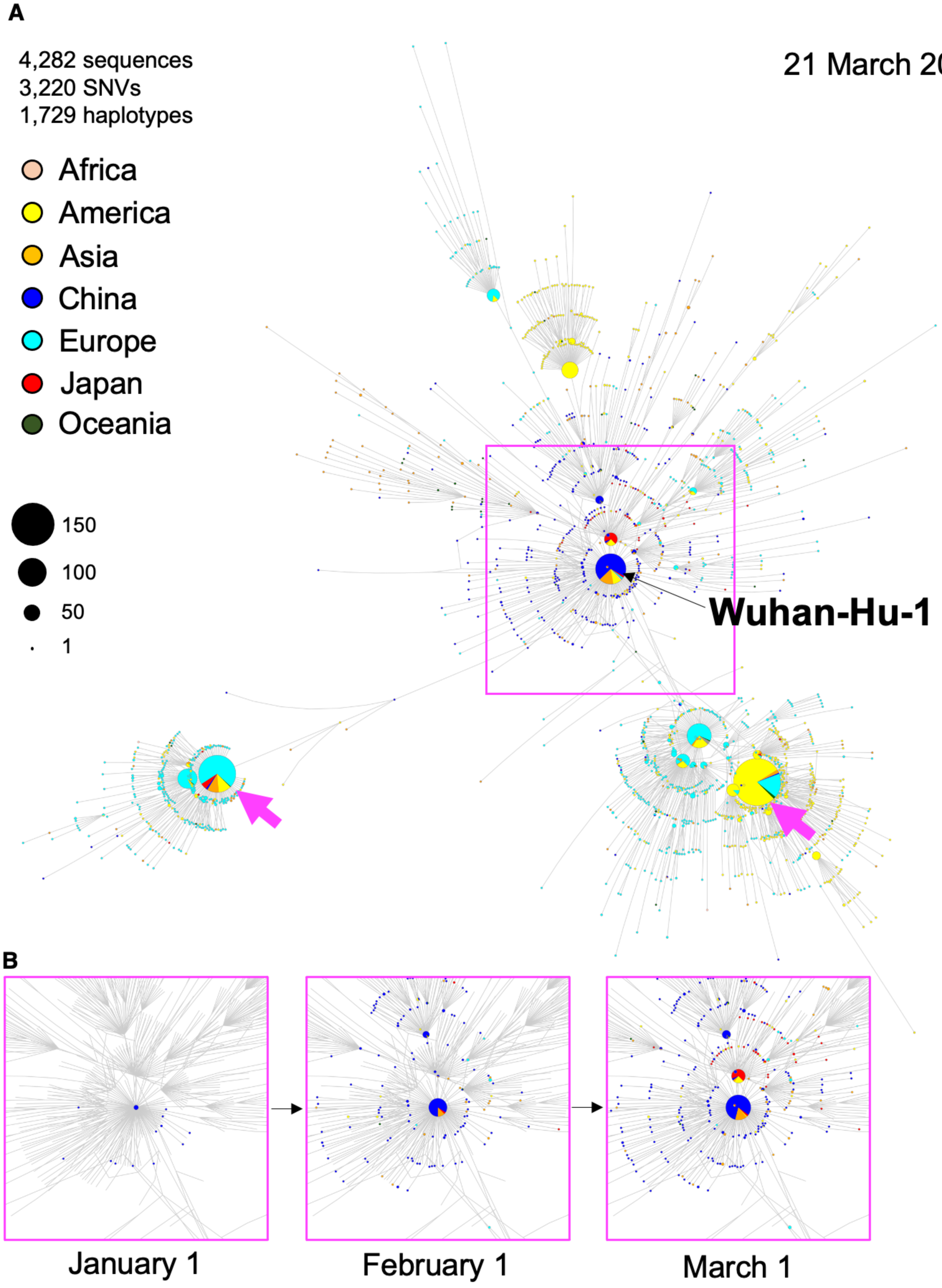

A
4,282 sequences
3,220 SNVs
1,729 haplotypes
21 March 2020
Africa
America
Asia
China
Europe
Japan
Oceania
150
100
50
1
Wuhan-Hu-1
B
January 1
February 1
March 1
